# Coinfection of *Aspergillus* and *Cryptococcus* in Immunocompromised Host: A Case Report and Review of Literature

**DOI:** 10.1155/2020/8888270

**Published:** 2020-07-24

**Authors:** Daniel W. Awari, Aditya S. Shah, Amber M. Sexton, Matthew A. Sexton

**Affiliations:** ^1^Mayo Clinic, Department of Anesthesiology and Perioperative Medicine, Rochester, MN, USA; ^2^Mayo Clinic, Department of Infectious Diseases, Rochester, MN, USA; ^3^Kentucky College of Osteopathic Medicine, Pikeville, KY, USA; ^4^Department of Critical Care, Rochester, MN, USA

## Abstract

Management of infections in the immunocompromised patient requires unique considerations that are not typically seen in the immunocompetent. Immunocompromised hosts require a broad set of differential diagnoses when presenting with febrile illness involving a wide variety of microbiology. Moreover, fungal infections are common, and cotreatment of fungal and bacterial infections occurs with regularity. Fungal coinfection, however, is rare. Here, we describe a patient with *Aspergillus* and recurrent *Cryptococcus neoformans* coinfection following completion of treatment for pulmonary cryptococcosis.

## 1. Introduction

It is well documented that patients who are immunocompromised are susceptible to a multitude of opportunistic organisms. Two of the more well-documented organisms that are clinically seen are *Aspergillus* and *Cryptococcus* species [1]. Both of these species individually have the capability of causing a primary infection of a single organ (typically lung) or rarely a disseminated infection causing multiorgan dysfunction [2, 3]. Concurrent infections among these two fungal species are even more uncommon. Current published literature comprises of only a very limited amount of case reports which describe cases in both immunocompetent and immunocompromised hosts [4–6]. Pending patient's clinical course, optimal therapy for these two species includes the triazoles [7–10]. Depending on the severity and patient's clinical state, combination therapy with these drugs and intravenous amphotericin has shown some benefits [11]. We present a case where an immunocompromised host developed a recurrent pulmonary *Cryptococcus neoformans* infection with new primary pulmonary aspergillosis after completion of a two-week intravenous amphotericin and ongoing fluconazole therapy.

## 2. Case Presentation

**Figure 1:**
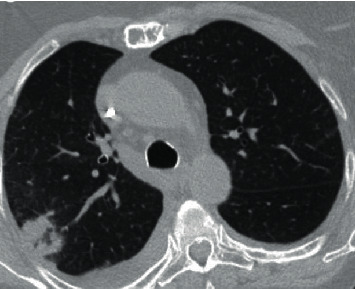
Initial chest CT showing right lobe infiltrate.

**Figure 2:**
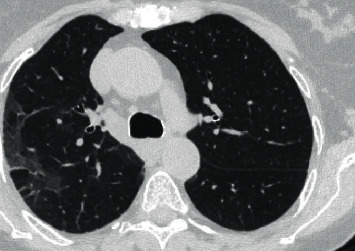
Repeat chest CT at three months showing interval improvement in right lobe infiltrate.

**Figure 3:**
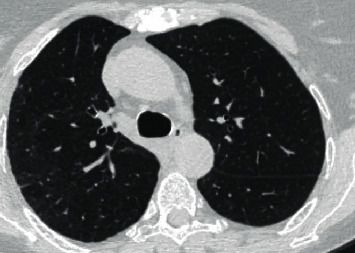
Repeat chest CT at six months shows continued improvement in right lobe infiltrate.

**Figure 4:**
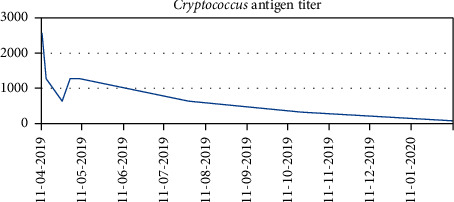
Cryptococcus antigen titers shown as dilutional ratios with higher numbers indicating more significant load. Titers can be seen decreasing over time indicating improvement in infection.

We present a case of a 69-year-old female with a past medical history of multiple myeloma followed by autologous peripheral stem cell transplant who was hospitalized after presenting with worsening dyspnea. Prior to her stem cell transplantation, she was found to have pulmonary *Cryptococcus neoformans* infection and was placed on oral flucytosine and intravenous liposomal amphotericin B. Subsequent studies revealed no central nervous system (CNS) involvement, and flucytosine was discontinued. She was discharged from the hospital to complete two weeks of intravenous liposomal amphotericin B and a year of oral fluconazole. She was readmitted after two weeks due to constitutional symptoms of fever, loss of appetite, and fatigue in addition to dry cough. *Cryptococcus* serum titers were 1 : 640 during this time as compared to 1 : 2560 at diagnosis. Bacterial and fungal cultures taken from peripheral and central access sites revealed no growth and *Aspergillus* serum antigen was negative. Chest CT revealed worsening pulmonary infiltrates in both the lingula and right upper lobe with a strong suggestion of fungal source (Figure 1). In this setting, a bronchoscopy with bronchoalveolar lavage was recommended. Concurrently, due to the expected low likelihood of dual infection with *Cryptococcus* and *Aspergillus*, empiric treatment of immune reconstitution syndrome was considered but held until invasive fungal infection was definitively ruled out. Subsequently, bronchoalveolar lavage was performed which showed an *Aspergillus* galactomannan antigen level of 3.258, confirming pulmonary aspergillosis, while cultures revealed no growth. Oral fluconazole was switched to oral voriconazole to provide additional coverage. Upon initiation of oral voriconazole, the patient defervesced and remained afebrile for the remainder of her nine-day hospital stay. She was subsequently discharged from the hospital after experiencing improvement in her condition and was diagnosed with pulmonary aspergillosis coinfection in the setting of pulmonary *Cryptococcus neoformans*. Repeat chest CT at three months (Figure 2) and six months (Figure 3) from hospitalization shows significant interval improvement of both infiltrate and effusion and outpatient *Cryptococcus* serum antigen titers showed a continual decrease from discharge (Figure 4). The patient was continued on oral voriconazole treatment for the duration of a year and tolerated the treatment well.

## 3. Discussion

Coinfection of *Aspergillus* and *Cryptococcus* is an uncommon event when one considers symptomatic primary fungal infection itself is rare. However, in certain populations, the risk of primary fungal infections is high [12]. In an immunocompromised patient experiencing recurrent fevers, if appropriate coverage of antibiotics does not yield defervescence and symptom resolution, then one should consider additional coverage with antifungals. While the patient was on fluconazole, it is not a first-line recommendation for the treatment of invasive aspergillosis. Voriconazole and liposomal amphotericin B are the currently recommended antifungals for the treatment of invasive aspergillosis [9]. Despite the low likelihood, it is imperative for the provider to ensure coinfection is not missed. It may be necessary to withhold treatments that can exacerbate infectious disease burden until infectious etiologies are ruled out, e.g., immune reconstitution inflammatory syndrome. In the above case, while providers were considering immune reconstitution syndrome, treatment for this disorder was held until other, less common, infectious causes of fever were ruled out. Ultimately, this proved to be a concurrent fungal infection, and implementation of the treatment for immune reconstitution syndrome would have significantly worsened this patient's fungal burden, likely leading to a poor outcome.

Previously described cases seen in the literature describe cases in both immunocompetent and immunocompromised patients, although immunocompromised patients are larger in number. These cases [4, 5] describe invasive therapies in order to diagnose the disease including video-assisted thoracic surgery to perform biopsies or lobectomies. These more invasive techniques were required due to the failure of less invasive diagnostic modalities like bronchoscopy. The above-described case was successfully diagnosed via bronchoscopy; however, more invasive diagnostic modalities must be considered if coinfection remains a possible diagnosis. Were coinfection not accurately diagnosed, systemic fungal infections with their associated morbidity would have developed.

Ultimately, diagnosis is paramount in order to successfully treat the disease. Without an accurate diagnosis, appropriate treatment cannot be initiated. Other case reports [4–6] describe pharmacologic therapies—fluconazole and itraconazole—that are not strongly recommended by the most recent IDSA guidelines. Currently, the primary treatment of invasive pulmonary aspergillosis is voriconazole [9], with other therapies—liposomal amphotericin B and isavuconazole—as alternatives. The initial therapy in this patient—long-term oral fluconazole following liposomal amphotericin B for two weeks following initial hospital discharge—was utilized for pulmonary cryptococcosis; however, this was unsuccessful in treating the invasive aspergillosis. The patient remained febrile and symptomatic and it was not until conversion to voriconazole did the patient improve. One must utilize the most current therapeutic recommendations to ensure treatment is optimal.

## 4. Conclusion

Febrile immunocompromised patients can often present with complicated infectious disease etiologies. Broad differential diagnoses must be maintained in order to determine the underlying cause that is otherwise uncommon in the immunocompetent. While standard diagnostic modalities are useful as shown in the case above, unfortunately, they may fail to provide a diagnosis and more invasive modalities must be considered and utilized [4, 5]. Most importantly, utilizing current recommendations provides optimal treatments for disease based on recent evidence. Suboptimal treatments may lead to ineffectual clearance of infection or allow for dual infection to occur. Coinfection of *Aspergillus* and *Cryptococcus* is a rare occurrence but can cause significant morbidity if undiscovered. Clinicians should maintain a high index of suspicion in immunocompromised patients and perform the diagnostic testing necessary to ensure this unlikely coupling is not missed.

## Data Availability

No data were used to support this study.
